# Nuclear factor of activated T-cell isoform expression and regulation in human myometrium

**DOI:** 10.1186/s12958-015-0086-0

**Published:** 2015-08-04

**Authors:** Evonne C. Chin-Smith, Frances R. Willey, Donna M. Slater, Michael J. Taggart, Rachel M. Tribe

**Affiliations:** Division of Women’s Health, King’s College London, Women’s Health Academic Centre KHP, St Thomas’ Hospital, 10th Floor, North Wing, Westminster Bridge Road, London, SE1 7EH UK; Physiology and Pharmacology, Cumming School of Medicine, Alberta Children’s Hospital Research Institute for Child and Maternal Health, University of Calgary, Alberta, T2N 4 N1 Canada; Institute of Cellular Medicine, Newcastle University, Newcastle-upon-Tyne, NE2 4HH UK

**Keywords:** A23187, Uterine smooth muscle, NFAT, Stretch, Transcription factors

## Abstract

**Background:**

During pregnancy, myometrial gene and protein expression is tightly regulated to accommodate fetal growth, promote quiescence and ultimately prepare for the onset of labour. It is proposed that changes in calcium signalling, may contribute to regulating gene expression and that nuclear factor of activated T-cell (NFAT) transcription factors (isoforms c1-c4) may be involved. Currently, there is little information regarding NFAT expression and regulation in myometrium.

**Methods:**

This study examined NFAT isoform mRNA expression in human myometrial tissue and cells from pregnant women using quantitative PCR. The effects of the Ca^2+^ ionophore A23187 and *in vitro* stretch (25 % elongation, static strain; Flexercell FX-4000 Tension System) on NFAT expression were determined in cultured human myometrial cells.

**Results:**

Human myometrial tissue and cultured cells expressed NFATc1-c4 mRNA. NFATc2 gene expression in cultured cells was increased in response to 6 h stretch (11.5 fold, *P* < 0.001, *n* = 6) and calcium ionophore (A23187, 5 μM) treatment (20.6 fold, *P* < 0.001, *n* = 6). This response to stretch was significantly reduced (90 %, *P* < 0.001, *n* = 10) in the presence of an intracellular calcium chelator, BAPTA-AM (20 μM).

**Conclusions:**

These data suggest that NFATc2 expression is regulated by intracellular calcium and *in vitro* stretch, and that the stretch response in human myometrial cells is dependent upon intracellular calcium signalling pathways. Our findings indicate a potentially unique role for NFATc2 in mediating stretch-induced gene expression *per se* and warrant further exploration in relation to the mechanisms promoting uterine smooth muscle growth in early pregnancy and/or labour.

## Background

It is widely accepted that a rise in the availability of intracellular Ca^2+^, through the up-regulation of calcium influx and release pathways, underpins the development of forceful uterine contractions during labour [1–3]. However, there is little understanding of how changes in calcium signalling in response to mechanical signals could drive changes in gene and protein expression in myometrium during early pregnancy or prior to labour to influence uterine development, growth and/or contractility.

Over the course of pregnancy, the myometrium must grow in size to accommodate the fetus and amniotic fluid, resulting in a 20-fold expansion in size and at term must become contractile in preparation for labour [4]. Animal models have shown that myometrial cells undergo a program of differentiation phases consisting of an early proliferative phase during which cells are hyperplastic, an intermediate synthetic phase consisting of cell hypertrophy, a third phase at around mid-gestation during which myometrial cells stop growing, contractile mechanisms are up-regulated and pro-quiescent factors are down-regulated and a final phase at the end of gestation in which cells switch to being highly active and committed to labour [5–8]. Studies that have measured uterine weight at opportune times in human pregnancy indicate that the human myometrium undergoes a period of rapid growth between the non pregnant state and the 20^th^ week of pregnancy and subsequently, and in contrast to animals, continues to grow at a slower rate until close to term [9, 10].

The link between exogenous signals such as mechanical stretch and endocrine signals and intracellular events to drive human uterine smooth muscle cell hyperplasia in early-mid pregnancy and hypertrophy in human myometrium are not clearly defined. In other cell types and smooth muscles, however, intracellular calcium signals are well known to regulate gene expression and growth through downstream activation and modulation of transcription factors [11–14] but in human pregnant myometrium, there is little documented about the role of Ca^2+^-sensitive transcription factors.

An important family of Ca^2+^-sensitive transcription factors are the nuclear factor of activated T-cells (NFAT) which consist of 4 isoforms, nuclear factor of activated T-cells, cytoplasmic, calcineurin-dependent 1 (NFATc1), NFATc2, NFATc3 and NFATc4. This group of transcription factors also include the Ca^2+^/calcineurin-insensitive NFAT5. NFAT protein activation is initiated by the phosphatase calcineurin, which is controlled by Ca^2+^ and calmodulin [15, 16]. Activated calcineurin dephosphorylates cytoplasmic NFAT proteins allowing rapid translocation of NFAT to the nucleus [16, 17] and binding to DNA. Other nucleoproteins can bind cooperatively with NFAT, including members of the AP-1 transcription factor [18].

NFAT transcription factors were originally identified to play a role in immune cell function, but have since been shown to be present in smooth muscle cell types, including human myometrial artery smooth muscle [19], mouse ileal smooth muscle [20] and mouse cerebral artery smooth muscle [21] and have been implicated in the regulation of genes that control cell cycle progression, cell development, cell differentiation and angiogenesis [22–26].

There are very few reports of NFAT expression in relation to myometrium. Tabata et al. [27] identified gestational changes in NFAT isoform c1, c3 and c4 mRNA expression in pregnant mouse uterus and more recently; Pont et al. [28] demonstrated oxytocin-induced NFAT nuclear translocation which mirrored pulses of oxytocin in a frequency-dependent manner in non-pregnant human myometrial cells.

The aim of this study was to identify NFAT isoforms in pregnant human myometrial tissue and primary myometrial cells. The regulation of NFAT mRNA expression by intracellular calcium was also investigated, as there are reports in other cell types that NFATc1 gene expression is up-regulated by rises in intracellular Ca^2+^ [29–34]. As mechanical strain/stretch, pregnancy and labour associated stimulus for uterine smooth muscle hyperplasia and hypertrophy may increase intracellular calcium [35–38] we also explored the effect of *in vitro* stretch on myometrial NFAT expression.

## Methods

### Subjects

Human myometrial biopsies were obtained at Caesarean section with informed written consent and institutional Ethics Committee approval (Guy’s and St Thomas’ Hospital NHS Trusts, London, UK; Office of Medical Bioethics, University of Calgary). Biopsies from the upper edge of the lower segment incision were obtained from pregnant women at the time of elective caesarean section (at term prior to labour), none of the women had underlying medical conditions (reasons for elective caesarean section at 37–40 weeks were: maternal request, breech presentation, previous caesarean section, fetal cardiac anomaly detected antenatally, stress incontinence, previous 3^rd^ degree tear or placenta praevia). In addition, lower segment human myometrium was also obtained from four groups of women at the time of caesarean section (LSCS) under the conditions of preterm no labour (PTNL; 29.2 ± 1.7 weeks’, myometrium samples from *n* = 5 women, reasons for elective caesarean section were intrauterine growth restriction or preeclampsia/pregnancy induced hypertension), preterm with labour (PTL; 30.0 ± 2.1 weeks’, samples from *n* = 4 women, reasons for caesarean section were breech presentation, previous caesarean section and increased fetal heart rate detected during labour), term no labour (TNL; 39.5 ± 0.4 weeks’, samples from *n* = 6 women, reason for caesarean section was previous caesarean section or breech) and term with labour (TL; 39.1 ± 0.6 weeks’, samples from *n* = 6 women, reasons for caesarean section were presented in early labour and elected to have section, failure to progress and/or fetal distress in labour). Whole myometrial tissue was either snap frozen and stored at −80 °C or used immediately for cell culture.

### Cell culture

Myometrial cells were dispersed enzymatically from tissue biopsies as described previously from term non-labouring (TNL) women [39]. Briefly, small segments of myometrium were dissected and chopped into 1–2 mm^3^ pieces and incubated for 30–40 min in Dulbeccos modified Eagles medium (DMEM) containing 1 mg/ml collagenase 1A, 1 mg/ml collagenase XI plus 0.1 % bovine serum albumin (BSA), penicillin (50 units/ml), and streptomycin (50 mg/ml). Cells were dislodged using a Pasteur pipette and then filtered through a 45 μm sterile filter, and washed twice in DMEM containing 10 % fetal calf serum (FCS) by centrifugation (450 × *g* 5 min). The cell pellet was suspended in DMEM supplemented with 10 % FCS, penicillin (25 units/ml), and streptomycin (25 mg/ml). Primary myocytes were seeded in T25 culture flasks and incubated at 37 °C in a humidified atmosphere of 95 % air/5 % CO_2._ Routine immunofluorescent labeling of cells with alpha-actin and calponin monoclonal antibodies was routinely performed to verify the purity of myocyte cultures. After the first 2 days of culture, media was replaced with DMEM supplemented with 5 % FCS, penicillin (25 units/ml), and streptomycin (25 mg/ml). The medium was changed every 2 days until cells were ~80 % confluent. Cells were used for experimentation at passage 2 (P2) in order to have enough material for *in vitro* the stretch protocol.

### Exposure of human myometrial cells to A23187 Ca^2+^ ionophore treatment

Human myometrial cells (P2) were cultured in six well culture plates in 3 ml DMEM plus 5 % FCS (Corning) until approximately 80 % confluent. Following replenishment of media (24 h prior to experimentation, 5 % FCS), cells were exposed to A23187 Ca^2+^ ionophore (5 μM, Sigma-Aldrich, UK) or vehicle control (0.1 % dimethyl sulfoxide, Sigma-Aldrich, Gillingham, UK) for 6 or 14 h at 37 °C in a humidified atmosphere of 95 % air/5 % CO_2_. At the end of the experiment, cells were rinsed with phosphate-buffered saline (PBS) and collected for RNA/protein extraction.

### Exposure of myometrial cells to tonic mechanical strain

A method similar to that used in the present has been described previously by us and others [40, 41]. Pregnant human myometrial cells (P2) were cultured in six well flexible-bottom culture plates pre-coated with collagen type I (Flexcell International Corp., Hillsborough, USA) in 3 ml DMEM plus 5 % FCS until approximately 80 % confluent. Media was replaced 24 hours before cells were subjected to 25 % tonic mechanical stretch for 6 h using a strain unit (Flexercell FX-4000 Tension system, Flexcell International Corp., Hillsborough, USA) housed in a cell culture incubator (37 °C, 95 % air, 5 % CO_2_). Time matched control cells were grown on the same flexible-bottomed culture plates, but were not stretched. In order to test the impact of buffering of intracellular Ca^2+^ whilst stretching, cells were pre-incubated for 1 h with BAPTA-AM (20 μM, Sigma-Aldrich, Gillingham, UK) or vehicle control (0.1 % dimethyl sulfoxide) before commencement of the 6 h tonic mechanical strain protocol. Subsequent to treatments, cells were washed with PBS and RNA extracted.

### RNA isolation from human myometrial tissue and human myometrial cells

Total RNA was extracted from human myometrial tissue from term non-labouring (TNL) women (~30 mg per sample) was homogenized using a TissueLyser (Qiagen, Crawley, UK) in Trizol® (Invitrogen, Paisley, UK) as per manufacturer’s recommendations. RNA was extracted from cultured myometrial cells using the RNeasy mini kit (Qiagen, UK) according to the manufacturer’s instructions. RNA samples were quantified using a Thermo Scientific NanoDrop ND-1000 spectrophotometer (Labtech, International Ltd, East Sussex, UK). The absorbance of each sample at 260 nm was used to calculate RNA concentration and an optical density 260/280 ratio was calculated to determine RNA purity. An OD 260/280 ratio of more than 1.8 was considered acceptable [42].

### Quantitative real-time PCR

**Fig. 1:**
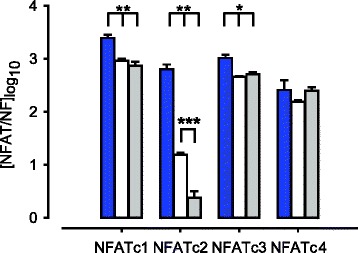
NFAT isoform expression profiles in pregnant human myometrial tissue and cultured cells. NFAT isoform mRNA expression in human myometrial tissue (blue bars, *n* = 6), primary (P0) human myometrial cells (white bars, *n* = 6) and cultured (P2) human myometrial cells (grey bars, *n* = 6). Data are expressed as mean ± SE log copy number [normalised to GeNorm normalisation factor (NF)]. Comparison of NFAT isoform expression in myometrial tissue *versus* P0 human myometrial cells and myometrial tissue *versus* P2 human myometrial cells (* *P* < 0.05, *** *P* < 0.001). Comparison of NFATc2 isoform expression in P0 human myometrial cells *versus* P2 human myometrial cells (*P* < 0.001)

**Fig. 2:**
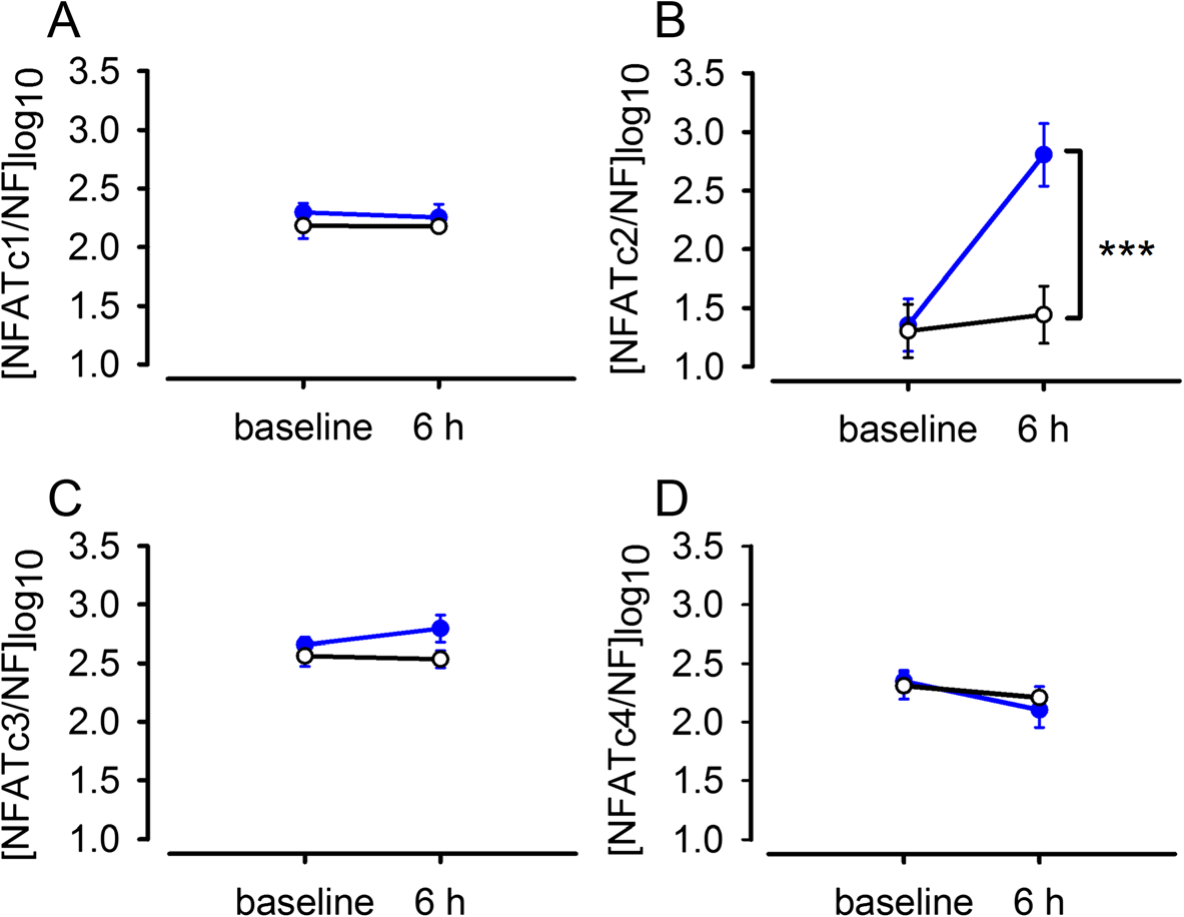
Effect of Ca^2+^ ionophore A23187 (5 μM) treatment on NFAT gene expression in pregnant human myometrial cells. Expression of NFAT isoforms in cultured (P2) human myometrial cells at 0 h (baseline) and exposed to A23187 (blue line) for 6 h and in vehicle control (0.1 % dimethyl sulfoxide) treated cells (black line). Data are expressed as mean ± SE log copy number [*n* = 6 cultures from myometrium biopsies taken from 6 pregnant women undergoing caesarean section at term, normalised to GeNorm normalisation factor (NF)]. Comparison between change in NFAT expression in A23187 and vehicle control treated cells from baseline to 6 h (*** *P* < 0.001)

cDNA was synthesised from 500 ng RNA using 0.25 μg random hexanucleotide primers (Promega, Southampton, UK) and 200 IU Superscript III (Invitrogen, Paisley, UK). Quantitative real-time PCR was performed with the use of SYBR Green chemistry (Bioline, London, UK) and a RotorGene 6000 Sequence Detector (Qiagen, Crawley, UK) using primer sets as listed in Table I. The PCR cycling conditions were as follows: an initial denaturation step of 95 °C for 10 min, then 45 cycles of a programme consisting of denaturation (95 °C, 15 s), annealing (60 °C, 30 s) and extension (72 °C, 20 s). Melt curve analysis confirmed a single product, and all products were sequenced to confirm identity. Negative reverse transcription reactions were used routinely to assess genomic DNA contamination. Test samples were run in duplicate in parallel with cDNA standards of known gene copy number abundance (10^6^ to 10^1^ copies). Cycle threshold values were used for analysis and abundance data was obtained for test samples by the generation of a standard curve based on the quantified cDNA. Abundance data for NFAT isoform expression for cell studies, Figures 1, 2, 4 and 5 were expressed relative to the geometric mean of a panel of housekeepers (glyceraldehyde 3-phosphate dehydrogenase (GAPDH), β-actin and β-2 microglobulin) as determined by GeNorm software [43] and for tissues only GAPDH was used due to limited cDNA availability and previous assessment which showed GAPDH was the most stable housekeeper for tissue analysis (Fig. 6). All data were log_10_ transformed.

### Preparation of whole cell protein lysates

Myometrial whole cell lysates were prepared by removing culture medium and rising twice with ice-cold PBS. The cells were then aspirated to dryness and 100 μl of lysis buffer (10 mM of Hepes-KOH [pH 7], 1 mM of dithiothreitol, 1 % nonident-P40, and protease-inhibitor cocktail [COMPLETE tablets; Boehringer-Mannheim Biochemicals, Lewes, Sussex, U.K.]) was added to each well and incubated on ice for 5 min. Cells were then scraped and the lysate removed and placed in to a 1.5 ml microfuge tube and centrifuged for 1 minute at 13,000 rpm to remove any cell debris. Tissue and whole cell lysate protein content was quantified using a Thermo Scientific NanoDrop ND-1000 spectrophotometer. Lysates were then diluted 1:1 with x 2 Laemmli sample buffer (Sigma) then boiled at 95 °C for 5 min. Samples were then stored at −20 °C until required, when they were thawed, then re-boiled for 5 min at 95 °C and centrifuged at 12470 g for 1 min prior to electrophoresis.

### SDS-PAGE and Western Immunoblotting

Whole cell lysate proteins were separated using 10 % Tris-Glycine precast gels (Invitrogen) using the XCell *SureLock*™ Mini-Cell system (Invitrogen). Following electrophoresis, proteins were transferred to Immobilion™-P transfer membrane (Millipore) using the XCell *SureLock*™ Mini-Cell blotting module wet transfer blotting system. After transfer of proteins to the membrane, non-specific sites were blocked by soaking the membrane in 100 % methanol for 10 s and then allowing the membrane to dry completely for approximately 15 min. Membranes were then incubated for at least 5 h with maximum speed of agitation at room temperature or overnight at 4 °C with the appropriate NFATc2 primary antibody (Abcam: AB2722) diluted 1:1000 or β-actin (Abcam: AB6276) diluted 1:5000 in tris-buffered saline with tween (TBS-T: 50 mM Tris, 150 mM NaCl, 0.2 % (v/v) Tween-20, pH 7.4) containing 10 % (w/v) BSA. Membranes were then washed for 3 × 20 min in TBS-T. Following the incubation of the membrane in a 1:10,000 dilution in TBS-T of horseradish peroxidase (HRP)-conjugated goat anti-mouse secondary antibody (BD Pharmigen) for 45 min, the membrane was washed a further 3 × 20 minutes in TBS-T. Immunoreactive proteins were visualised using enhanced chemiluminescence (ECL™) (Amersham) according to the manufacturer’s instructions. Densitometric quantification of immunoreactive bands was carried out using BioRad Molecular Quantity One software, version 4.4.0. To verify equal protein loading, blots were also probed with β-actin antibody.

### Statistical analysis

Data were analysed using Stata 10.0 (Stata Corp., College Station, USA). Differences between NFAT isoform expression in tissue and cells were assessed using linear regression. Results were expressed as ratios of the group means with 95 % confidence intervals; mean gene expression ± SEM is displayed graphically. The impact of experimental interventions on gene expression in human myometrial cells over time was estimated using linear regression on the log-copy number with a fixed subject effect to adjust for clustering and interaction tests were carried out where necessary. Mean fold changes over time in gene expression are presented in the text with 95 % confidence intervals; mean gene expression ± SEM at each time point is displayed graphically. Significance for all data was taken at *P* < 0.05. ‘n’ refers to the total number of cell cultures (each originating from a biopsy from a different subject).

## Results

### NFAT mRNA expression in human myometrial tissue, primary human (P0) myometrial cells and cultured (P2) human myometrial cells

NFAT mRNA for all isoforms (c1-c4) was detected in human myometrial tissue (*n* = 6). NFAT isoforms c1-c4 were also expressed in P0 (*n* = 6) and P2 (*n* = 6) human myometrial cells, but NFATc1, NFATc2 and NFATc3 expression was suppressed in P0 and P2 human myometrial cells compared to myometrial tissue. NFATc2 expression was lower in P2 compared to P0 cells (95 % CI: 71.4 to 91.7, *P* < 0.001). (Fig. 1).

### Effect of Ca^2+^ ionophore A23187 treatment on NFAT gene expression and protein in human myometrial cells

**Fig. 3:**
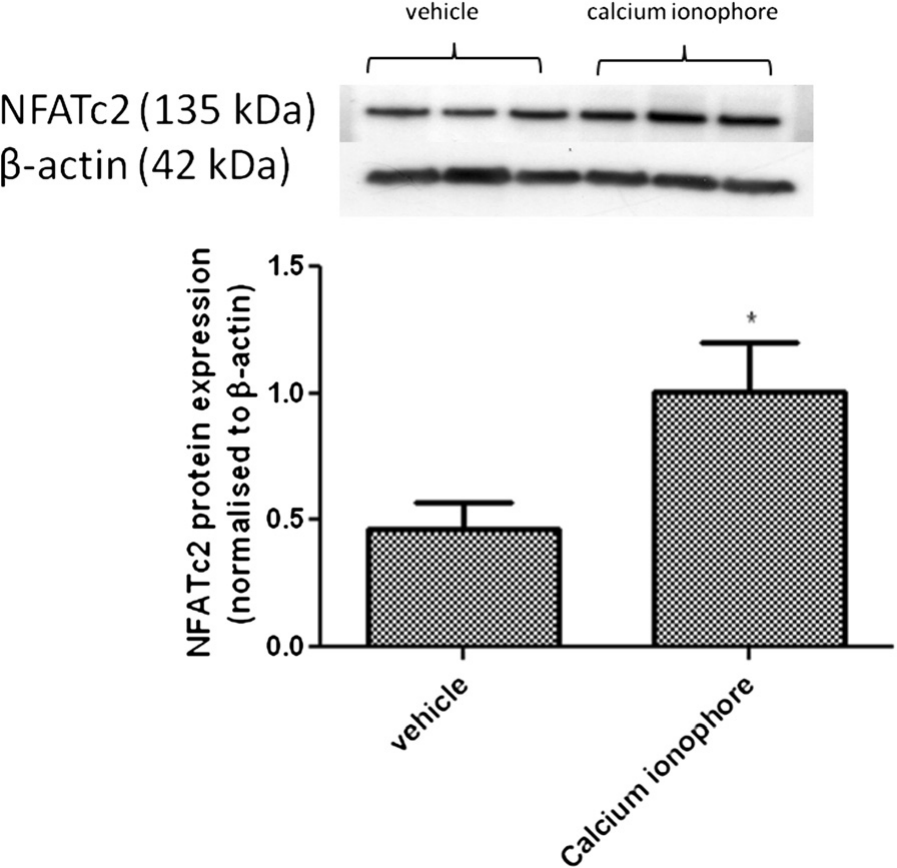
Effect of Ca^2+^ ionophore A23187 (5 μM) treatment on NFATc2 protein expression in pregnant human myometrial cells. P2 human myometrial cells were exposed to A23187 for 14 h. Representative immunoblots are shown for NFATc2 and β-actin protein expression. Data are expressed as mean ± SE arbitrary units normalised to β-actin protein expression, *n* = 6. *P < 0.05 compared to vehicle control

Application of the Ca^2+^ ionophore A23187 caused a 20.6 times (95 % CI: 7.6 to 55.6, *P* < 0.001, *n* = 6) increase in NFATc2 gene expression over 6 h compared to vehicle control (Fig. 2). There was no significant effect of Ca^2+^ ionophore A23187 treatment on NFATc1, NFATc3 or NFATc4 expression. Incubation of human myometrial cells with Ca^2+^ ionophore A23187 for 14 h significantly increased NFATc2 protein expression (*p* < 0.05, *n* = 6) (Fig. 3).

### Effect of *in vitro* stretch on NFAT gene expression in human myometrial cells

**Fig. 4:**
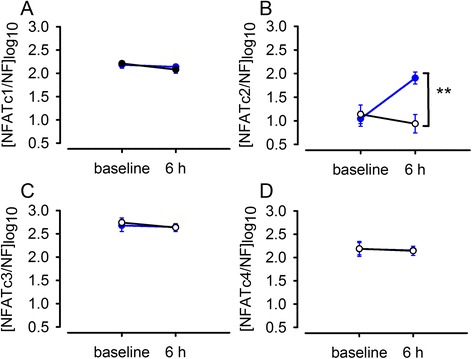
Effect of *in vitro* stretch on NFAT gene expression in pregnant human myometrial cells. Expression of NFAT isoforms in cultured (P2) human myometrial cells at 0 h (baseline) and exposed to 25 % *in vitro* strain (blue line) for 6 h and in non-stretched time-matched control cells (black line). Data are expressed as mean ± SE log copy number [*n* = 6, normalised to GeNorm normalisation factor (NF)]. Comparison between change in NFAT expression in stretched and non-stretched cells from baseline to 6 h (* *P* < 0.001)

*In vitro* stretch of cultured (P2) human myometrial cells caused an 11.5 times (95 % CI: 4.3 to 30.9, *P* < 0.001, *n* = 6) increase in NFATc2 gene expression over 6 h compared to non-stretched cells (Fig. 4). There was no effect of *in vitro* stretch on the expression of any other of the NFAT isoforms.

### Impact of BAPTA-AM on NFATc2 gene expression in stretched human myometrial cells

**Fig. 5:**
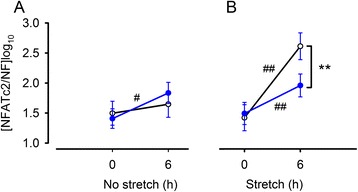
Impact of BAPTA-AM on NFATc2 gene expression in cultured (P2) pregnant human myometrial cells during a 6 h stretch period. A) NFATc2 expression in non-stretched control cells (t = 0 to t = 6 h) treated with BAPTA-AM (20 μM) (blue line) or vehicle control (0.1 % dimethyl sulfoxide) (black line). B) NFATc2 expression in 6 h stretched (25 % static strain) cells (t = 0 to t = 6 h) treated with BAPTA-AM (blue line) or vehicle control (black line). Data are expressed as mean ± SE log copy number [*n* =10, normalised to GeNorm normalisation factor (NF)]. In non-stretched cells there was a slight change in NFATc2 expression (# *P* < 0.05) from t = 0 h to t = 6 h in BAPTA-AM treated cells, but there was no significant difference in BAPTA-AM-treated *versus* vehicle control at t = 6 h. Both BAPTA-AM treated and vehicle control treated cells under stretch demonstrated a significant change in NFATc2 expression between t = 0 h to t = 6 h (##, *P* = 0.001) but the effect of stretch was significantly greater in cells in the absence of BAPTA-AM (** *P* < 0.001)

There was a small effect (1.9 fold, 95 % CI: 1.3 to 2.7, *P* < 0.05, *n* = 10) of BAPTA-AM on NFATc2 expression in non-stretched cells (change from *t* = 0 h to *t* = 6 h) but not in the vehicle control (Fig. 5), but there was no difference between absolute NFATc2 mRNA expression in BAPTA-AM treated cells compared to cells exposed to vehicle at the 6 hour time point. NFATc2 gene expression was significantly increased 11.0 times (95 % CI: 4.6 to 26.4, *P* < 0.001, *n* = 10) in stretched cells, but there was a 90 % reduction (95 % CI: 72.3 % to 96.4 %, *P* < 0.001, *n* = 10) in the effect of stretch on NFATc2 gene expression in the presence of BAPTA-AM.

### NFATc2 mRNA expression in preterm and term human myometrial tissue

**Fig. 6:**
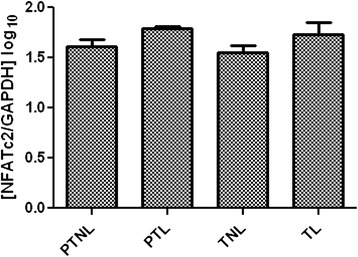
Effect of gestation and labour on NFATc2 gene expression in human myometrial tissue. Data are expressed as mean ± SE log copy number normalised to GAPDH. PTNL = 5, PTL = 4, TNL = 6, TL = 6

NFATc2 mRNA expression was detected in myometrial tissues taken from women delivering preterm and term gestations. There was no significant difference in tissues taken from women prior to or during labour (Fig. 6).

## Discussion

This study demonstrates the presence of mRNA for all Ca^2+^ /calcineurin-dependent NFAT isoforms c1-c4 in pregnant human myometrium and cultured cells. This is in good agreement with previously a published report of NFAT isoform expression in non-pregnant human myometrial cells [28]. However, the expression profile in human myometrial tissue contrasts with NFAT expression profiles in other smooth muscle tissue types, including human myometrial artery, mouse ileal smooth muscle and mouse cerebral artery smooth muscle in which NFATc2 has not been detected [19–21]. Moreover, although NFATc2 has been detected in smooth muscle from human and rat aorta [44–46] as well as human myometrial arteries after culture [19], the presence of NFATc2 in pregnant myometrial and cultured cells indicates a potentially important role for NFATc2 in human myometrium.

All four isoforms, NFATc1-4, were expressed in human myometrium tissue taken from women at term, but prior to labour onset. In contrast, cultured (P0) human myometrial cells displayed an altered NFAT mRNA expression profile compared to tissue, with NFATc1, c2 and c3 isoform expression significantly decreased in primary cells that were cultured in media containing 5 % serum. NFATc1 and NFATc3 gene expression remained stable following a further period of culture (until P2), but NFATc2 expression decreased further. The differences between tissue and primary cells NFATc expression levels could reflect the multicellular composition of myometrial tissue (e.g. immune cells, vascular cells etc.) but alternatively, the reduction in NFATc1, NFATc2 and NFATc3 gene expression is more likely to be due to phenotypic changes induced by the cell culture environment, and indicative that NFAT genes can be modulated in human myometrial cells. In our culture systems, cells are grown in the presence of 5 % serum and receiving signals to promote hyperplasia, whereas in myometrial tissue at term cells are no longer dividing [8].

Interestingly, the human myometrium cell culture (P2) associated reduction in NFATc2 mRNA and protein expression was reversed by exposing the cells to myometrial cells to Ca^2+^ ionophore A23187. This is of interest as whilst NFATc1 has been previously demonstrated in immune cells to be susceptible to gene regulation, NFATc2 mRNA and it associated protein has only been found to be constitutively expressed in other tissues [30, 32, 34]. The explanation for this tissue-difference in isoform regulation is unclear due to limited information on NFAT promoter behaviour, but may reflect the dependency of NFATc1 and NFATc2 isoform mRNA regulation on additional factors that are specific to different cell types.

Our findings also show that prolonged *in vitro* mechanical stretch increased NFATc2 expression *in vitro* after 6 h. This should be investigated to see whether it occurs at other time-points. Mechanical stretch has been implicated as a signal for mediating gene expression prior to labour onset; a mechanism for promoting contraction-associated proteins [47], but is also a signal for promoting cellular proliferation and growth [48–50]. Our data, therefore, could indicate a contribution of NFAT regulated transcription in human myometrial tissue during pregnancy particularly at earlier gestations when cells are proliferating (as per our *in vitro* stretch model). However, it is noted that in the small number of human myometrium tissues studied NFATc2 mRNA expression remained relatively stable at preterm and term gestations prior to and after labour onset, and so the involvement of this isoform in mediating changes associated with labour cannot be inferred without further study. It is interesting to note, that in mice NFATc1, 2 and 4 mRNAs have been reported to increase during pregnancy, although these data were obtained in an oxytocin receptor knock mouse model and the mRNA data were not quantified [27].

The observation that stretch induced changes in NFATc2 expression are suppressed by BAPTA-AM further implies that changes in intracellular calcium in response to stretch are important in this response. The impact on NFATc2 related protein expression still requires examination. Under non-stretch conditions, NFATc2 mRNA also increased slightly in the BAPTA-AM treated cells over time, but the expression level at 6 h was no different from the vehicle control at this time point, suggesting that the change was not biologically significant.

If these *in vitro* findings reflect responses *in vivo* then it is possible that uterine stretch (or other pregnancy associated stimuli that induce increases in Ca^2+^) could contribute to induction of NFATc2 expression and/or activation in myometrium in pregnant women. The translation and functional impact of these Ca^2+^ and/or stretch induced responses in myometrium now require further confirmation at the protein level. Ideally, studies looking at NFAT protein expression (and the other isoforms) and localisation in human tissues at different stages of gestation, and in cells, would shed further light other potential functional contributions NFTAc2 or other isoforms make in regulating uterine smooth muscle growth and contractility in pregnancy. We experienced difficulties with using antibodies to distinguish different NFATc isoforms (data not shown), which limited the scope of our study.

Investigations into the relationship of NFAT regulation with other important myometrial transcription factors, for example, NF-κB [51–55] and AP-1 would also be of interest given their reported interactions in other cell types [56–61].

## Conclusion

In summary, the study presents novel data concerning NFAT mRNA expression and regulation by calcium in human myometrium. Our findings also indicate a potential role for NFATc2 in mediating stretch-induced gene expression *per se*. These warrant further exploration in relation to the mechanisms promoting myometrial cell proliferation/growth in pregnancy and contractility in prior to labour. In particular a more focussed study of NFATc2 protein function and translocation, using confocal imaging with immunohistochemistry and live cell calcium sensitive dyes, in response to different intracellular calcium and mechanical stretch signals is required.
